# TREM2 deficiency in microglia accelerates photoreceptor cell death and immune cell infiltration following retinal detachment

**DOI:** 10.1038/s41419-023-05735-x

**Published:** 2023-03-28

**Authors:** Wenchuan Zhou, Yutong Zhou, Jincan He, Yuqing Rao, Ping Fei, Jing Li

**Affiliations:** Department of Ophthalmology, Xinhua Hospital Affiliated to Shanghai Jiao Tong University School of Medicine, Shanghai, 200092 China

**Keywords:** Cell death in the nervous system, Apoptosis, Cell death and immune response

## Abstract

Retinal detachment (RD) occurs in several major retinal conditions and often causes irreversible vision loss due to photoreceptor cell death. Retinal residential microglial cells are activated following RD and participate in photoreceptor cell death via direct phagocytosis and the regulation of inflammatory responses. Triggering receptor expressed on myeloid cells 2 (TREM2) is an innate immune receptor exclusively expressed on microglial cells in the retina, and has been reported to affect microglial cell homeostasis, phagocytosis and inflammatory responses in the brain. In this study, increased expression of multiple cytokines and chemokines in the neural retina was observed starting at 3 h following RD. Trem2 knockout (*Trem2*^*−/−*^) mice exhibited significantly more photoreceptor cell death than wild-type controls at 3 days after RD, and the number of TUNEL positive photoreceptor cells progressively decreased from day 3 to day 7 post-RD. A significant thinning of the outer nuclear layer (ONL), with multiple folds was observed in the *Trem2*^*−/−*^ mice at 3 days post-RD. Trem2 deficiency reduced microglial cell infiltration and phagocytosis of stressed photoreceptors. There were more neutrophils in *Trem2*^*−/−*^ retina following RD than in controls. Using purified microglial cells, we found Trem2 knockout is associated with increased CXCL12 expression. The aggravated photoreceptor cell death was largely reversed by blocking the CXCL12-CXCR4 mediated chemotaxis in *Trem2*^*−/−*^ mice after RD. Our findings suggested that retinal microglia are protective in preventing further photoreceptor cell death following RD by phagocytosing presumably stressed photoreceptor cells and by regulating inflammatory responses. TREM2 is largely responsible for such protective effect and CXCL12 plays an important role in regulating neutrophil infiltration after RD. Collectively, our study pinpointed TREM2 as a potential target of microglial cells to ameliorate RD-induced photoreceptor cell death.

## Introduction

Retinal detachment (RD) occurs in several major ocular conditions including diabetic retinopathy, age-related macular degeneration (AMD), and ocular injury [1–4]. Aging and myopia, especially high myopia, are also common causes of RD [5, 6]. RD causes the physical separation of the photoreceptor cell outer segment from the underlying retinal pigment epithelium (RPE), and is followed by profound pathologic changes of the neuroretina [7, 8]. The death or functional loss of photoreceptor cells is the major consequence of RD, which occurs as fast as 12 h following RD [8, 9]. Depending on the anatomical location and the extension of detachment, RD often leads to irreversible and significant vision loss [10].

Inflammation plays important roles in mediating the pathologic changes following RD [11]. Increased secretion of proinflammatory cytokines, such as CCL-2, TNF-α, IL-1β, and IL-6, were detected in both animal models of RD and the vitreous samples of RD patients [12–15]. A critical role of mostly Müller glia-derived CCL-2 in mediating photoreceptor cell death following RD was reported. Mice with genetic deficiency of *Ccl2* showed substantially less TUNEL^+^ photoreceptor cells after RD [13]. In a more recent study using similar approaches, the role of increased IL-6 on monocyte chemotaxis and activation following RD was reported [16]. However, the changes of proinflammatory cytokines and chemokines immediately following RD remain unclear.

Microglia, the residential immune cells of the retina, play important roles in maintaining retinal homeostasis and integrity [17]. In healthy eyes, microglia have small cell body and highly ramified processes, and are located in the plexiform layers and ganglion cell layer. Following RD, microglia transform into ameboid shape with retracted processes, migrate to the injured sites and perform multiple functions which are both photoreceptor protective and photoreceptor offensive, such as the production of inflammatory factors, phagocytosis of the stressed photoreceptor cells and the recruitment of peripheral immune cells [16, 18–20]. It was shown that the depletion of microglia increased TUNEL^+^ photoreceptor cells, and reduced the infiltration of CD11b^+^ cells following RD [20]. The results suggest that phagocytic activity of microglia toward photoreceptor cells following RD is of protective nature to prevent more photoreceptor cells from dying. However, the molecular mechanism which mediate microglia cell phagocytosis in the retina is unknown, and the role of microglia-mediated leukocyte infiltration on RD-induced photoreceptor death remains unclear.

Triggering receptor expressed on myeloid cells 2 (TREM2) is a single-pass transmembrane immune receptor that is almost exclusively expressed on the surface of microglia in the central nervous system (CNS) and retina [21, 22]. TREM2 signaling regulates microglial cell survival, metabolism, cytoskeleton remodeling, phagocytosis, and inflammatory responses [23–27]. TREM2 is also needed for stress-induced activation of microglial cells in the brain. TREM2-deficient microglia in brain showed impaired phagocytosis of apoptotic neurons and increased expression of pro-inflammatory factors [26]. Based on its functions in brain microglial cells, we hypothesized that TREM2 in retinal microglial cells mediates the phagocytosis of photoreceptor cell and may also affect cytokine production thus regulate the leukocyte chemotaxis following RD.

To test this hypothesis, we compared the progression of RD-induced inflammatory responses and photoreceptor death in normal C57BL/6J (WT) and Trem2 knockout (*Trem2*^*−/−*^) mice. We analyzed the progressive changes of retinal transcriptional profile during the first 24 h after RD. We found that Trem2 deficiency caused impaired microglial migration and phagocytosis, accelerated peripheral immune cell infiltration, and aggravated photoreceptor cell death following RD. We also found that Trem2-deficient microglia express higher level of CXCL12, and blocking the binding of CXCL12 to CXCR4 could effectively protect photoreceptors from RD-induced cell death. These observations suggested that the net protective role of microglia in RD is mediated via TREM2 signaling.

## Materials and methods

### Animals husbandry and the induction of RD

All animal experiments were conducted following the guidelines of the Association for Research in Vision and Ophthalmology Statement for the Use of Animals in Ophthalmic and Vision Research and were approved by the Institutional Animal Care and Use Committee of Xinhua Hospital Affiliated to Shanghai Jiao Tong University School of Medicine. C57BL/6J (WT) mice were purchased from Shanghai Jihui Animal Care Co., Ltd. (China), and *Trem2*^*−/−*^ mice on the C57BL/6J background were purchased from Shanghai Model Organisms Center Inc. (Shanghai, China). These animals were allowed free access to water and chow, and were housed in a specific-pathogen-free mouse facility under 12-h light/12-h dark cycle. All mice used for experiments were 8–10 weeks old.

Subretinal injection of sodium hyaluronate is a widely accepted approach to create RD [7]. Mice were anesthetized with intraperitoneal injection of a mixture of tiletamine-zolazepam (40 mg/kg body weight) and xylazine (10 mg/kg body weight), and pupils were dilated with 0.5% tropicamide. The nasal conjunctiva at the posterior limbus was incised and separated from the sclera. A scleral tunnel was made by a 32-gauge needle, followed by corneal puncture to lower intraocular pressure. Subsequently, we inserted a 34-gauge needle with syringe into the subretinal space and injected 1% sodium hyaluronate gently to detach 60% of the neural retina from the underlying RPE. Finally, conjunctiva was reattached with surgical glue (Vetbond). Eyes with subretinal hemorrhage or unsuccessful detachment were excluded. No randomization was performed. Investigator was blinded while assessing the severity of RD since genotype was carried out by another investigator.

Plerixafor (AMD3100, HY-10046 from MedChemExpress, NJ, USA), a CXCR4 antagonist, was dissolved in phosphate buffer solution (PBS) at 10 mg/mL and injected (1 μL) at a single dose into the subretinal space after RD induction.

### Retinal tissue bulk sequencing and data analysis

The transcriptional profiles of WT and *Trem2*^*−/−*^ retina at different times after RD were generated by bulk sequencing. Total amount and integrity of RNA were assessed using the RNA Nano 6000 Assay Kit of the Bioanalyzer 2100 system (Agilent Technologies, CA, USA). Library was prepared and qualified from each sample. Different libraries were pooled according to the effective concentration and the target amount of data off the machine, and sequenced using Illumina NovaSeq 6000. The sequencing data were normalized, and the differentially expressed genes (DEGs) (|log_2_Fold Change|>1 and adjusted *p* value < 0.05) were identified using R package “DESeq2”. Venn diagram analysis was used for the extraction of overlapped genes, and Metascape (http://metascape.org) was used for functional enrichment analyses, including Gene Ontology and Kyoto Encyclopedia of Genes and Genomes enrichment analyses. We collected and grouped terms with an enrichment factor >1.5, minimum count of 3, a *p* value < 0.01 into clusters. Time series analysis of DEGs was conducted using R package “Mfuzz”. The Fuzzy c-means clustering is a soft clustering method performed using the Mfuzz algorithm with two key parameters (*c* = number of clusters and *m* = fuzzification parameter).

We also downloaded the sequencing data (GSE28133) from GEO database. GSE28133 included 19 human retinal samples from RD patients and 19 control samples without RD. The platform used to obtain these data was GPL570 (Affymetrix Human Genome U133 Plus 2.0 Array). A similar data analysis procedure as described above was performed.

### Retinal tissue dissociation, microglial cell purification, and flow cytometry

WT and *Trem2*^*−/−*^ mice were euthanized by sodium pentobarbital (120 mg/kg body weight) and their eyes were enucleated. Retinas were dissected in oxygenated Ames solution and incubated at 37 °C for 25 min in enzyme mix (Neural tissue dissociation kit, Miltenyi Biotec, #130-094-802). The digestion was stopped by adding Ames solution containing 5% BSA. The mixture was further dissociated by gentle pipetting to obtain single-cell suspension.

The CD11b^+^ microglial cells in the single-cell suspension were isolated using the magnetic CD11b MicroBeads (Miltenyi Biotec, #130-093-634) and the matching column (Miltenyi Biotec, #130-042-401). To increase the purity of microglial cells, we used two columns sequentially for each preparation.

For flow cytometry, the above single-cell suspension was filtered through a 70-mm strainer, washed with PBS containing 1% FBS, and stained with LIVE/DEAD dye and antibodies (CD45, CD11B and Ly6G) for 30 min at 4 °C according to the manufacturer’s instructions. Flow cytometry data were analyzed with FlowJo software.

### Immunohistochemistry and TUNEL labeling of retinal sections

Immunohistochemistry of frozen tissue sections was conducted as previously described [28]. After enucleation, the eyecup without lens was fixed in 4% paraformaldehyde for 1 h at room temperature, sequentially dehydrated in 20% and 40% sucrose at 4 °C, embedded in optimal cutting temperature compound and then sectioned at 10 μm thickness using a microtome (CM1950, Leica Biosystems, Wetzlar, Germany).

Tissue sections were blocked with 10% goat serum and permeabilized with 0.2% Triton X-100 for 2 h at room temperature, and incubated with primary antibodies overnight at 4 °C. Primary antibodies used included rabbit anti-IBA1 (Wako, #019-19741, 1:500), rat anti-CD68 (Bio-rad, #MCA1957, 1:100), rabbit anti-Ly6G (Servicebio, #GB11229, 1:200) and rabbit anti-cleaved caspase 3 (Cell Signaling, #9664, 1:200). Alexa Fluor-488-conjugated goat anti-rabbit, Alexa Fluor-568-conjugated goat anti-rabbit and Alexa Fluor-568-conjugated goat anti-rat secondary antibodies were used. The slides were sealed with antifade reagent containing DAPI (ThermoFisher, #S36938).

The terminal deoxynucleotidyl transferase dUTP nick end labeling (TUNEL) was performed according to the manufacturer’s specifications (Roche, #11684795910). Stained retinal sections were imaged with fluorescent microscopy (Olympus BX51) or confocal microscopy (Leica TCS SP8). The retinal sections stained with hematoxylin and eosin were imaged with light microscopy. We measured five points of outer nuclear layer (ONL) thickness on each section with same spacing distance, and calculate the average thickness of each eyeball.

### RNA extraction, reverse transcription, and relative quantitative PCR (qPCR) analysis

TRIzol reagent (Thermo Fisher Scientific, Waltham, MA, USA) in combination with RNA Clean & Concentrator™-5 (ZYMO Research, Irvine, CA, USA) was used for RNA extraction in this study. PrimeScript™ RT reagent kit (Takara, Japan) was used for reverse transcription. TB Green® Premix Ex Taq ™ II (Takara, Japan) was used for qPCR analysis. Gene expression levels relative to Gapdh for protein-coding RNA were calculated using the 2^−ΔΔ^Ct method. The primer sequences are listed in (Additional file 1: Table S1).

### Statistical analysis

Sample size was chosen considering at least three independent experiment. Unpaired Students’ *t* test or two-way analysis of variance was used for statistical analyses with GraphPad Prism 8.0.2 (GraphPad Software Inc., San Diego, CA, USA). The data are presented as mean ± standard deviation. Statistical significance was accepted at *p* < 0.05.

## Results

### Early inflammatory responses with increased cytokine and chemokine expression following RD

**Fig. 1:**
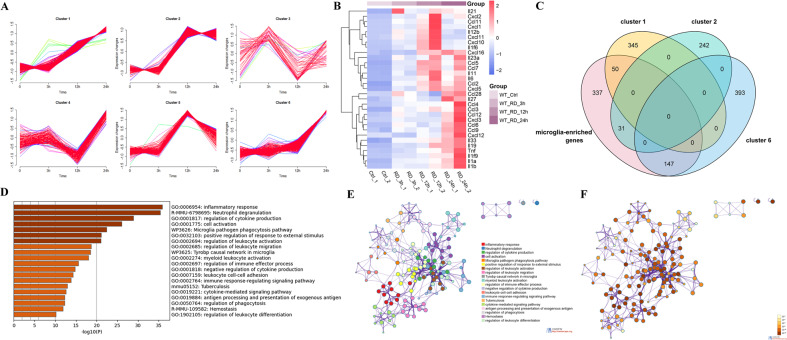
Early inflammatory responses with increased cytokine and chemokine gene expression following RD. **A** Time-course transcriptional profiles of up-regulated DEGs in WT mouse retina within 24 h after RD. **B** Heatmap of inflammatory cytokine and chemokine expression in the up-regulated DEGs. **C** Venn diagram showing the overlapped genes among Cluster 1, Cluster 2, Cluster 6, and microglia-enriched genes. **D** The top 20 GO terms and pathways over-represented by genes of Cluster 6. **E** Network of enriched terms colored by cluster ID, where nodes that share the same cluster ID are typically close to each other. **F** Network of enriched terms colored by *p* value, where terms containing more genes tend to have a more significant *p* value.

Most of the studies published so far focused on retinal reactions 24-h and later after RD. There is a lack of information about the molecular events occurring immediately after RD. To fill the gap, we generated the transcriptional profile of the retina at 0, 3, 12, and 24 h following RD (Additional file 1: Table S2). The changes of gene expression along the time course were analyzed using Mfuzz time clustering. The upregulated DEGs between time-points were separated into six clusters according to their temporal expression patterns (Fig. 1A).

Functional analysis of the genes in each cluster showed that Cluster 1, 2, and 6 were mainly involved in inflammatory and immune responses, such as the secretion of cytokines, regulation of immune effector process, and the regulation of leukocyte migration (Additional file 1: Table S3–S5). Gene expressions in Cluster 1 and 2 increased significantly starting at 3 h after RD, while genes in Cluster 6 increased significantly starting at 12 h after RD. Cluster 4 are enriched with ribosomal protein coding genes, which showed a quick decrease between 3 and 12 h after RD, following by steep increase, suggesting cell damage induced the interruption of protein translation at early time point, and a quick response to increased demand of likely a new set of genes in response to RD (Additional file 1: Table S6). Genes in Cluster 5 were involved in the regulation of defense response and cellular response to interferon (Additional file 1: Table S7). Cluster 3 contains a group of loosely organized genes which are enriched in complement activation keratin sulfate degradation and cell death (Additional file 1: Table S8). We identified the major cytokines and chemokines in the upregulated DEGs and analyzed the changes of their expression (Fig. 1B). The results showed three temporal patterns of gene expression among these cytokines: cytokines that reached peak expression at 12 h after RD and decreased afterwards, as represented by *Cxcl1*, *Cxcl10*, and *Cxcl11*; cytokines that reached peak expression at 12 h and remained high, as represented by *Ccl2*, *Ccl5*, *Ccl7,* and *Il6*; cytokines that reached peak expression at 24 h, as represented by *Tnf*, *Il1a*, *Il1b*, *Il33*, and *Cxcl12*.

In an attempt to dissect the contribution of microglial cells in the early inflammatory responses, we compared the genes in the above clusters with a panel of 565 microglia-enriched genes obtained from literature [29] (Additional file 1: Table S9). Using Venn diagram analysis, we identified overlapped genes in Cluster 1, 2, and 6. Cluster 6 contained the highest number of microglia-enriched genes (147 overlapped genes) compared with Cluster 1 and 2, suggesting a similar transcriptional profile associated with microglia (Fig. 1C). We then performed functional enrichment analysis using Metascape on genes in Cluster 6 and found that they were enriched mainly in GO: 0006954 (inflammatory response), R-MMU-6798695 (Neutrophil degranulation), GO: 0001817 (regulation of cytokine production), GO: 0001775 (cell activation) and WP3626 (Microglia pathogen phagocytosis pathway) (Fig. 1D–F). The results again supported the participation of microglial cell in early inflammatory responses following RD, and suggested that microglia are actively associated with leukocyte migration, activation and phagocytosis.

We also performed the same analysis on down-regulated DEGs of the time-course transcriptional profiles. (Additional file 1: Table S10). They were grouped into four clusters according to their temporal expression patterns (Additional file 2: Fig. S1A). Functional enrichment analysis of the genes in each cluster showed that Cluster 1 was mainly enriched in cellular response to interferon-beta, Cluster 2, 3, and 4 were enriched in neural retina-specific signal transduction functions, such as visual perception, action potential, neuron projection development, inorganic ion transmembrane transport and trans-synaptic signaling (Additional file 2: Fig. S1B–G). These findings suggested that acute RD led to immediate damage in retinal neuron communication and visual signal transduction.

### Microglial infiltration and phagocytosis of photoreceptor cell in the ONL after RD

**Fig. 2:**
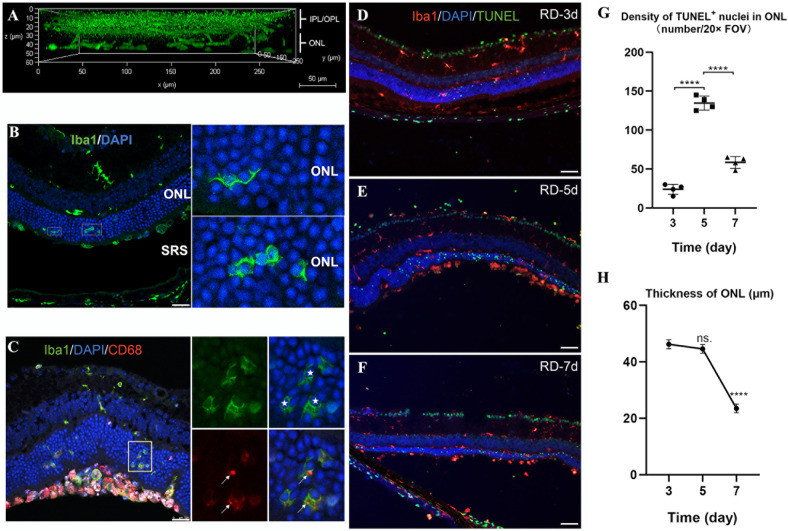
Microglial infiltration and phagocytosis of photoreceptor cells in the ONL after RD. **A** Side view of 3D-constructed images showing that microglia migrate to the injured ONL at 3 d post-RD. Scale bar, 50 μm. **B** Infiltrating microglia terminate in a specialized cup-like structure and extend around photoreceptor somata. Scale bar, 25 μm. **C** CD68 co-localizes to IBA1^+^ infiltrating microglia and peripheral immune cells. Scale bar, 25 μm. Solid white stars highlight phagocytosed neurons, and arrows point to phagocytic microglia. **D–F** Co-immunolabeling for TUNEL^+^ nuclei and IBA1^+^ cells at 3, 5, and 7 d post-RD. Scale bar, 50 μm. Quantification of the number of TUNEL^+^ cells (**G**, as density of TUNEL^+^ nuclei per 20× Field of view) and ONL thickness (**H**) at 3, 5, and 7 d post-RD. (*n* = 4 animals at each time point). Data points and error bars indicate mean ± SD. ns, not significant. *p < 0.05, **p < 0.01, ***p < 0.001, and ****p < 0.0001.

Next, we investigated the infiltration and phagocytic activities of retinal microglia following RD. Retinas were isolated for whole-mount immunohistochemistry at 3 d post-RD and stained for IBA1. The entire retinal thickness was scanned by confocal microscopy. The side view of 3D-reconstructed images showed microglia at the injured ONL (Fig. 2A). Immunofluorescent staining of IBA1 on cross-sectioned 3 d post-RD retinal tissue revealed migrating microglial cells with radially oriented processes. The processes of the migrating microglia often formed specialized cup-like structures and further extended around photoreceptor cell somata (Fig. 2B). These cells also express phagocytosis marker CD68, a lysosome-associated membrane protein, as indicated by immunofluorescent staining. These results suggested the phagocytosis of the infiltrating microglia. Cells at the detached subretinal space also showed expression of CD68 and IBA1 (Fig. 2C). Further analysis showed that they were mostly positive of Ly6G (Additional file 2: Fig. S2, discussed below), suggesting that they were recruited neutrophils.

We also examined the thickness of the photoreceptor layer and the density of TUNEL^+^ nuclei (Fig. 2D–F). Quantitative analysis showed that the number of TUNEL + nuclei increased from day 3 to day 5 post-RD, and declined at day 7 while the ONL progressively thinned (Fig. 2G,H).

### TREM2 deficiency accelerates photoreceptor degeneration, and affects microglial cell infiltration and distribution following RD

**Fig. 3:**
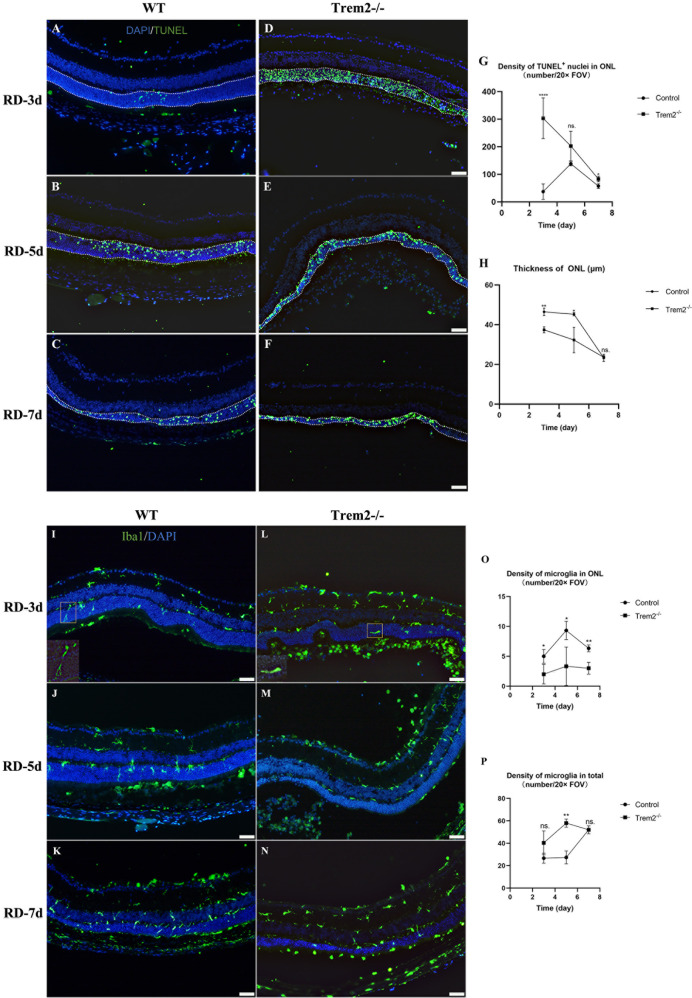
TREM2 deficiency accelerates photoreceptor degeneration, and affects microglial cell infiltration and distribution following RD. Representative immunofluorescent images of TUNEL^+^ nuclei from 3 d to 7 d post-RD in WT (**A**–**C**) and *Trem2*^*−/−*^ (**D**–**F**) mouse. Scale bar, 50 μm. **G**, **H** Comparison of the number of TUNEL^+^ nuclei and ONL thickness between WT and *TREM2*^*−/−*^ mouse retina at 3, 5, and 7 days post-RD. Representative immunofluorescent images of IBA1^+^ cells from 3 d to 7 d post-RD in WT (**I**–**K**) and *Trem2*^*−/−*^ (**L**–**N**) mouse. Scale bar, 50 μm. **O**, **P** Comparison of the infiltration and distribution of IBA1^+^ cells between WT and *Trem2*^*−/−*^ mouse retina at 3, 5, and 7 days post-RD. Data was curated and presented as described in Fig. 2. Three to six animals were used for each time point in each group.

To investigate the role of TREM2 on photoreceptor cell death, we induced RD in *Trem2*^*−/−*^ mice (Fig. 3A–F). Unlike the WT retina, *Trem2*^*−/−*^ retina showed significantly more TUNEL^+^ photoreceptor cells at day 3 post-RD. While the number of apoptotic photoreceptor cell reached a peak at day 5 post-RD in WT retina, the *Trem2*^*−/−*^ retina showed progressive reduction of TUNEL^+^ cells. The changes in TUNEL^+^ cells were also confirmed by immunofluorescent staining of cleaved caspase-3 in both WT and *Trem2*^−/−^ retina (Additional file 2: Fig. S3). Consistently, we observed significant thinning of the photoreceptor cell layer at day 3 post-RD in *Trem2*^*−/−*^ retina, which was not observed in WT RD (Fig. 3G, H). However, by day 7, there was no difference in the number of TUNEL^+^ cells or the thickness of the photoreceptor cell layer between WT and *Trem2*^*−/−*^ retina. Collectively, the results suggested that TREM2 deficiency accelerated and exacerbated photoreceptor cell death at early phase following RD.

Immunofluorescent staining of IBA1 staining showed less infiltrating microglial cell on day 3 post-RD in the ONL and subretinal space of *Trem2*^*−/−*^ mice. Furthermore, the microglia crawling in the ONL of *Trem2*^*−/−*^ mice lacked the extension of processes (Fig. 3I–N). Quantitative analysis showed that there were more IBA1^+^ cells in the retina of *Trem2*^*−/−*^ mice at 5 d post-RD than controls, further suggesting that a number of *Trem2*-deficient microglia were limited to the inner retina (Fig. 3O, P).

### TREM2 deficiency advances the peak time of inflammatory response and accelerates the immune cell infiltration

**Fig. 4:**
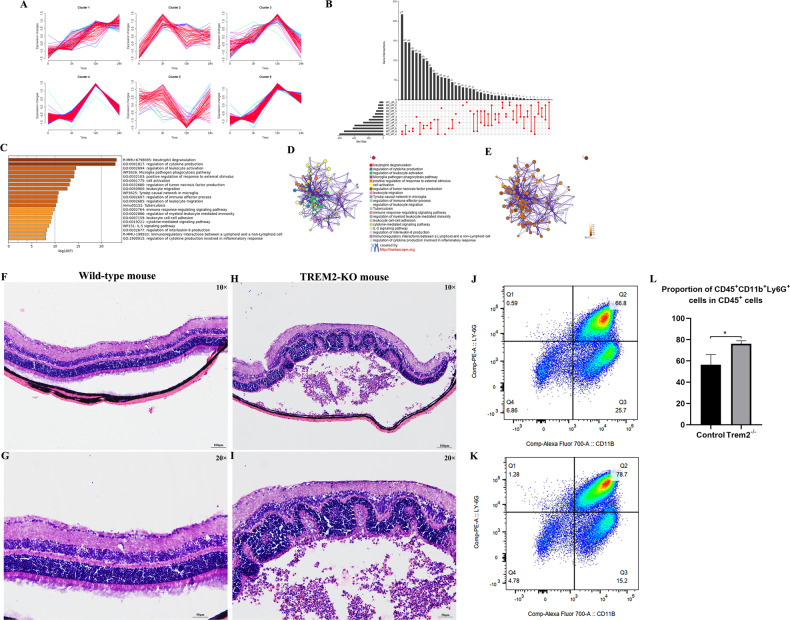
*Trem2* deficiency advances the peak time of inflammatory response and accelerates immune cell infiltration. **A** Time-course transcriptional profiles of up-regulated DEGs in *Trem2*^*−/−*^ mouse retina within 24 h after RD. **B** UpSet plot showing that Cluster 6 of WT and Cluster 4 of *Trem2*^*−/−*^ contain the most overlapped genes (218 out of 540), suggesting that the same genes are activated earlier in *Trem2*^*−/−*^ mouse following RD. **C** The top 20 GO terms and pathways over-represented by the 218 overlapped genes. **D** Network of enriched terms colored by cluster ID. **E** Network of enriched terms colored by *p* value. Representative histopathological images of WT (**F**, **G**) and *Trem2*^*−/−*^ (**H**, **I**) mouse after RD indicates that *Trem2*^*−/−*^ mice exhibit extensive retinal folds with numerous infiltrated neutrophils accumulation at the grooves. Scale bar, 100 μm (10×) and 50 μm (20×). Representative flow cytometry plots showing the gating strategy for neutrophils (CD45^+^CD11b^+^Ly6G^+^) in the WT (**J**) and *Trem2*^*−/−*^ (**K**) mouse after RD. **L** Quantification analysis of the flow cytometry indicates that a higher percentage of neutrophils was observed in *Trem2*^*−/−*^ mouse retina.

To examine the effect of *Trem2* deficiency on retinal inflammatory responses following RD, we obtained the transcriptomic profile of *Trem2*^*−/−*^ retina at 0, 3, 12, and 24 h after RD (Additional file 1: Table S11). Mfuzz time clustering of the Upregulated DEGs yielded six clustered (Fig. 4A). We analyzed the gene intersections of each cluster between WT and *Trem2*^*−/−*^ mouse. As shown in the UpSet plot, Cluster 4 of *Trem2*^*−/−*^ contained the most overlapped genes with Cluster 6 of WT mice (218 out of 540) (Fig. 4B). Similar to Cluster 6 in the WT, the genes in Cluster 4 of *Trem2*^*−/−*^ were also mostly enriched in inflammatory responses and phagocytosis as revealed by functional enrichment analysis (Fig. 4C–E). However, the expression of these genes was significantly increased at 12 h post-RD in *Trem2*^*−/−*^, while they were not increased until 24 h post-RD in WT controls. Therefore, the data suggested that Trem2 deficiency advanced the peak time of inflammatory responses.

The histopathological changes of retina from *Trem2*^*−/−*^ mouse RD model corroborated the gene expression changes. At 3 d post-RD, we observed extensive retinal folds in *Trem2*^*−/−*^ mice, with numerous infiltrated immune cell accumulation at the grooves (Fig. 4F–I). The cells with lobulated nucleus in the subretinal space were identified as neutrophils. Flow cytometry analysis also confirmed that there was a remarkable increase of neutrophils (CD45^+^CD11b^+^Ly6G^+^) in the total population of CD45^+^ cells in *Trem2*^*−/−*^ retina at 3 days post-RD compared to controls (76.1% v.s. 56.3%, *Trem2*^*−/−*^ v.s. WT) (Fig. 4J–L). Collectively, these data suggested that Trem2 deficiency accelerated inflammatory cell infiltration and led to more severe photoreceptor cell damage shortly after RD.

### TREM2 deficiency upregulates *Cxcl12* expression potentiating photoreceptor cell death

**Fig. 5:**
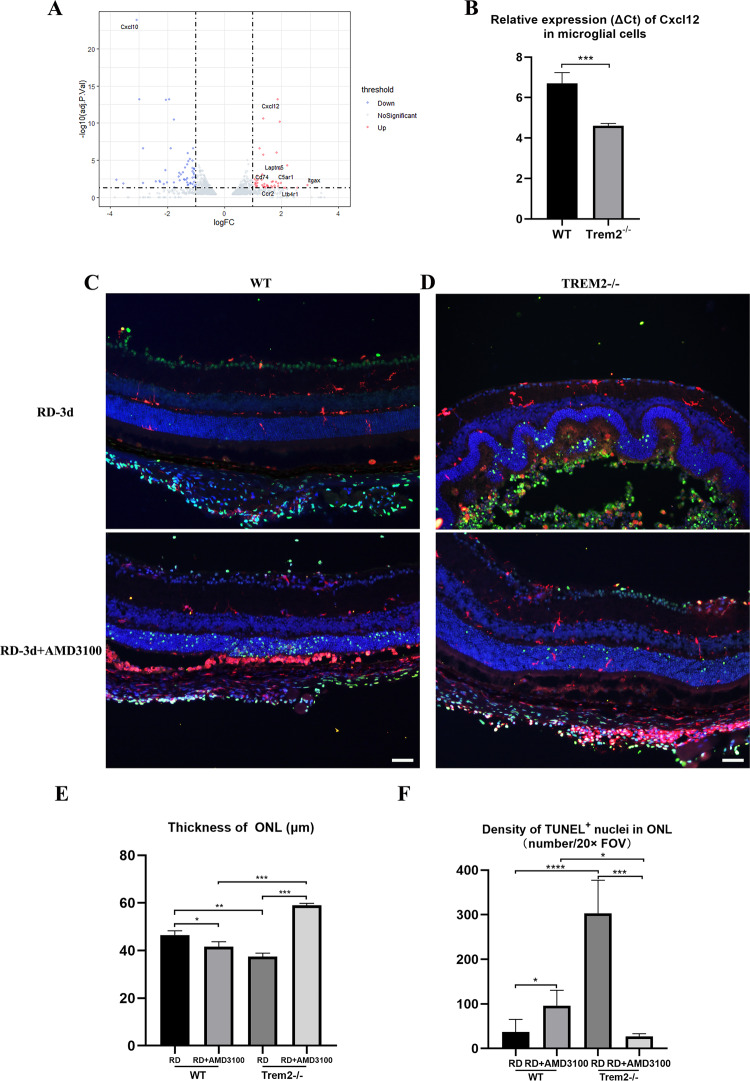
*Trem2* deficiency upregulates *Cxcl12* expression potentiating photoreceptor cell death. **A** Volcano plot showing that the expression level of Cxcl12 is increased at 12 h post-RD in *Trem2*^*−/−*^ mouse. **B** The expression of Cxcl12 is increased in *Trem*2-deficient microglial cells at resting state. Co-immunolabeling for TUNEL^+^ nuclei (green) and IBA1^+^ cells (red) at 3 d post-RD in WT (**C**) and *Trem2*^*−/−*^ (**D**) mouse treated with and without AMD3100. **E**, **F** Quantitative analyses demonstrate that compared to control groups, the AMD3100 treatment significantly reduced immune cell infiltration, the number of TUNEL^+^ cells in the ONL and the thinning of ONL at 3 d post-RD in the *Trem2*^*−/−*^ mice. Column heights and error bars indicate mean ± SD. ns, not significant, *p < 0.05, **p < 0.01, and ***p < 0.001.

To further explore the cause of accelerated inflammatory responses in *Trem2*^*−/−*^ mice following RD, we compared the whole retinal transcriptomic profile between WT and *Trem2*^*−/−*^ without RD and found that *Cxcl12* was the only protein coding gene which showed significant increased expression in the KO retina (log_2_FC = 1.10, adj. *p* = 0.029) (Additional file 1: Table S12). The differences in *Cxcl12* increased at 3 h (log_2_FC = 5.79, adj. *p* = 0.55) and 12 h post-RD (log_2_FC = 1.87, adj. *p* = 7.14E-14) (Fig. 5A). *Cxcl12* is expressed at low level in normal retina. To test if *Trem2* deficiency in microglial cells caused increased *Cxcl12* expression in untreated *Trem2*^*−/−*^ retina, we purified microglial cells from untreated WT and *Trem2*^*−/−*^ retina using magnetic beads coupled CD11b antibody and extracted total RNA. The purity of the cells was confirmed by the enrichment of microglial homeostatic genes *Cx3cr1* and *Tmem119* (Additional file 2: Fig. S4). Real-time PCR analysis showed a 3.3-fold (95% CI: 2.6–3.9) increase of *Cxcl12* in *Trem2*-deficient microglial cells (Fig. 5B).

CXCL12 is a major chemokine responsible for neutrophil recruitment via binding to CXCR4. As presented above, there was more neutrophil accumulation in *Trem2*^*−/−*^ retina following RD than controls. To test if increased expression of *Cxcl12* is responsible for accelerated photoreceptor degeneration in *Trem2*^*−/−*^ mice, we treated the WT and *Trem2*^*−/−*^ mice with AMD3100, a selective CXCR4 antagonist which inhibits CXCL12-mediated chemotaxis. AMD3100 was injected into the subretinal space immediately after sodium hyaluronate. The treatment increased the number of TUNEL^+^ cells and reduced the ONL thickness in WT RD model (Fig. 5C). In *Trem2*^*−/−*^ RD mice, the treatment preserved the ONL, and reduced TUNEL^+^ cells at ONL (Fig. 5D). Compared to WT RD retina, the AMD3100 treatment significantly reduced immune cell infiltration, number of TUNEL^+^ cells in the ONL and the thinning of ONL at 3 d post-RD in the *Trem2*^*−/−*^ mice (Fig. 5E, F). Quantification of TUNEL^+^ and Ly6G^+^ cells in *Trem2*^*−/−*^ RD retina showed simultaneous reduction of both cells after AMD3100 treatment (Additional file 2: Fig. S5). These data suggested that CXCL12-mediated recruitment of neutrophil cells was responsible for the severe photoreceptor cell damage observed in *Trem2*^*−/−*^ mice following RD.

### TREM2 is upregulated in human RD

**Fig. 6:**
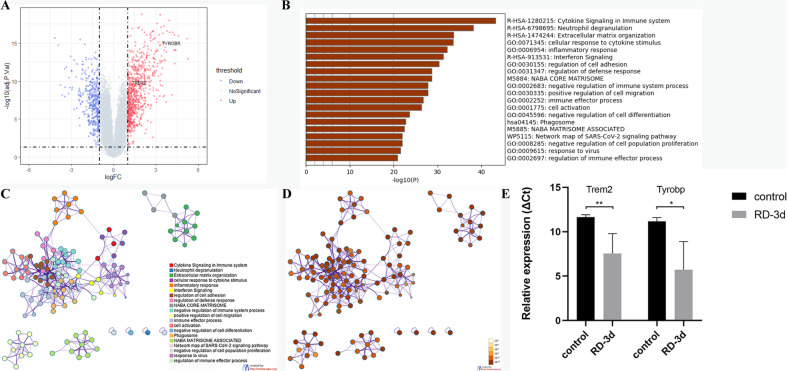
*TREM2* is upregulated after RD. **A** Volcano plot indicates that *TREM2* and its adapter *TYROBP* are upregulated in the retinal samples of RD patients. **B** The top 20 GO terms and pathways over-represented by the DEGs in RD patients. **C** Network of enriched terms colored by cluster ID. **D** Network of enriched terms colored by *p*-value. **E** Relative expression (ΔCt) of *Trem2* and *Tyrobp* genes in retina tissues 3 d post-RD. (*n* = 4 or more animals in each group). Column heights and error bars indicate mean ± SD. ns, not significant, *p < 0.05, and **p < 0.01.

Studies in the CNS showed that microglial cells with increased *TREM2* expression define a distinctive group of cells which exist in brains of neurodegenerative conditions. These cells are believed to play a net protective role [30]. Heterozygous rare variants in *TREM2* increase the risk for neurodegenerative diseases, such as Alzheimer’s disease, Parkinson’s disease and amyotrophic lateral sclerosis [31–34]. To test if RD is also associated with increased *TREM2* expression, we compared transcriptomic profiles of retinal samples taken from human with and without RD (GSE28133, as described in the Methods). This dataset included retinal samples from 19 RD patients and 19 controls. We found significant upregulation of *TREM2* and its adapter *TYROBP* in RD tissues (Fig. 6A). In order to obtain an overall picture of molecular events at the time points of *TREM2* upregulation, we identified the DEG between these two groups and performed functional enrichment analysis. We found the upregulated DEGS are mainly over-represented in R-HSA-1280215 (Cytokine signaling in immune system), R-HSA-6798695 (Neutrophil degranulation), R-HSA-1474244 (Extracellular matrix organization), GO: 0071345 (cellular response to cytokine stimulus) and GO: 0006954 (inflammatory response) (Fig. 6B–D). The results suggested ongoing inflammatory responses. Likewise, we found significant increase of both genes in mouse retina samples 3 days post-RD. (Fig. 6E). On average, the expressions of *Trem2* and *Tyrobp* were 35.74-fold (95% CI: −5.17−76.7) and 189.1-fold (95% CI: −64.39–442.5) higher in RD groups than in controls, respectively.

## Discussion

The pathologic changes following RD have been described in both human and animal models of RD [8, 12]. However, the molecular events immediately after RD are still incompletely understood. In this study, we found that the changes in retinal gene expression occur mainly between 3 and 12 h post-RD. These included the down regulation of genes involved in vision perception, synaptic communication and ion channels, and the upregulation of many cytokines and chemokines. The changes of the transcriptomic profiles also suggested that inflammatory response is a key driver of pathologic changes following RD, as indicated by previous studies [12, 35, 36]. Increased IL-6, CCL-2, TNF-α and IL-1β levels were found previously at a later time point after RD [12–15]. Studies have shown that IL-6 was mainly produced by macrophage migrated from the vitreal-retinal interface upon RD, and the signaling mediated by IL-6 receptor, gp130, was a major determinant of the severity of RD. CCL-2, mainly produced by Müller glia upon RD, also plays significant roles in propagating the inflammatory signals after RD. We found that *Il6* and *Ccl2* gene expression increased rapidly after RD, reached peak levels at 12 h and remained high. Unlike *Il6* and *Ccl2*, the expression of *Il1β* and *Tnf* increased relatively slowly but progressively and reached a peak at 24-h, suggesting that they are likely from different cellular sources. In addition, we found that the expression of several neural retinal cell-expressed chemokines, such as *Cxcl9* and *Cxcl10*, increased quickly and steeply at 12 h post RD, followed by a decline at 24 h. In a mouse model of optic nerve injury, upregulation of *Cxcl10* was found and the blocking of Cxcl10-mediated leukocyte infiltration significantly prevented retinal ganglion cell death [37]. Overall, the temporal features of individual cytokine expression provided us with clues in identifying players which may be important in propagating the inflammatory signals immediately after RD, and are worth further investigation.

RD is accompanied by the activation of microglial cells, which manifests by direct phagocytosis of photoreceptor cells and by regulating the inflammatory responses [20]. Previous studies suggested a protective role of microglial cell following RD as mice without microglial cells showed increased photoreceptor cell death [20]. However, the mechanism which mediate microglial cell activation following RD remains elusive. It is unknown whether the phagocytosis activity and the inflammatory responses are regulated through common or separate mechanisms. The present study supported the protective role of microglial cells following RD and further suggested that *Trem2* was involved in regulating both the phagocytic and inflammatory responses of microglial cells following RD, as *Trem2*^*−/−*^ mouse showed massive TUNEL^+^ photoreceptor cells, rapid neutrophil accumulation and accelerated thinning of ONL following RD, and *Trem2*-deficient microglia exhibited slower motility and reduced phagocytic activity. Mechanistically, we found that the increased *Cxcl12* expression in *Trem2-*deficient microglial cells is likely the direct cause of the exacerbated inflammatory responses.

In microglial cells of the brain, TREM2 has been shown to regulate cell migration, phagocytic activity and cytokine secretion [23–27]. *Trem2*-deficient microglia of the brain showed impaired clearance of apoptotic neurons [26]. In human *TREM2*-deficient iPSC-derived microglial cells, the lack of *TREM2* resulted in substrate-specific deficits in phagocytosis [38]. On the other hand, increased *Trem2* expression promoted microglial cell migration and phagocytic clearance of β-amyloid enriched extracellular deposits and degenerating neurons [39]. Our results are consistent with the reported TREM2 activity in brain microglial cells. We noticed that there were a few phagocytic microglial cells in the ONL of *Trem2*^−*/−*^ mouse retina following RD. This is not surprising as the phagocytic activity of microglial cells is regulated by multiple mechanisms such as phosphatidylserine-mediated receptors, complement receptors, purinergic receptors and toll-like receptors [40–43]. Furthermore, the phagocytic activity of microglial cell following RD is protective for the rest of the photoreceptors, since the lack or reduction of it was associated more photoreceptor cell death. This is presumably due to the fact that RD is an acute event which lead to immediate photoreceptor cell death, and the clearance of dead or dying cells helps prevent the release of pro-apoptotic molecules which would cause further cell death. However, the phagocytic activity of microglia toward stress yet not dead photoreceptor cells in rd10 mouse retina was shown to be detrimental, as the depletion of microglial cells prevented photoreceptor death and deterred the speed of retinal degeneration [44]. In fact, whether the effect of microglial phagocytosis on neurons is protective or detrimental has been discussed extensively in the brain [44–47]. It is generally believed that the specific features of the condition and the targeted cells are important factors which influence the overall effect of microglial phagocytosis. It would be interesting to investigate the involvement of TREM2 in the phagocytosis of photoreceptor cells in models of retinal degeneration.

The regulatory effect of TREM2 on cytokine expression was also reported in microglial cells of the brain. TREM2 deficiency led to increased expressions of IL-1β, TNF-α, NOS-2 and IL-6 [26, 48, 49]. Overexpression of TREM2 decreased expression of TNF-α, IL-1β, and NOS2 [26]. A recent study showed that TREM2 regulates the expression of the above cytokines in LPS-stimulated retinal microglia [50]. We found that *Trem2* deficiency led to increased *cxcl12* expression in microglial cells, an effect which was not reported before. We further demonstrated that Cxcl12 is likely the mediator which linked *Trem2* deficiency to the augmented neutrophil infiltration and exacerbated ONL thinning, as blocking the effect of CXCL12 largely reversed the phenotype. This is also consistent with the observation that photoreceptor cell death was even more exacerbated in the presence of *Trem2*-deficient microglia (the present study) than in the absence of microglial cell all together as observed in a previous study [20]. We believe that TREM2 has an inhibitory effect on *Cxcl12* expression in microglia and the absence of *Trem2* leads to increased *Cxcl12* expression. The significant upregulation of *Cxcl12*, but not other cytokines in resting *Trem2*-deficient microglia observed in this study demonstrated the condition-dependent effect of TREM2-mediated cytokine expression. Our results also expanded the repertoire of cytokines/chemokines that are critically involved in the inflammatory responses following RD.

A significant feature of TREM2 in microglia is its indispensable role in the activation of a distinctive group of microglia defined as disease-associated microglia (DAM) in the brain of neurodegenerative conditions at later stage [30]. Research in both human and animal models of brain neurodegenerative conditions suggested that DAM is overall protective by clearing β-amyloid enriched extracellular deposits [51–53]. TREM2 has been reported to bind lipoproteins, such as ApoE, LDL and CLU/apoj, which form complexes with Aβ aggregates, facilitating their uptake by microglia [54–56]. A group of activated microglial cells with similar gene expression profile was also reported in animal models of retinal degeneration [57]. We observed increased expression of *Trem2* and its partner gene *Tyrobp* at 3 days after RD in the present study. Increased *TREM2* and *TYROBP* expression were also found in retinal samples of human RD, in the context of active inflammatory responses. While the infiltration of monocytes may contribute to the increased *Trem2* expression in both cases, it is possible that there is activation of DAM-like microglial cells at later stage of RD. Exploring the activity of these cells may help in better re-establish the fine connections among photoreceptor cells, retina pigment epithelial cells and other retinal neurons which are required for functional recovery after RD [58].

## Conclusions

In conclusion, the present study demonstrated a significant contribution of TREM2 in the net protective role of microglial cells following RD via the regulation of the phagocytic and inflammatory activities. We also identified CXCL12 is an important mediator for microglia-mediated neutrophil chemotaxis. Our results suggested that modulation of TREM2 activity in retinal microglial cells may be able to control photoreceptor cell damage immediately after RD.

## Supplementary information

Additional file 1

Additional file 2

## Data Availability

The data supporting the findings of this study are available from the corresponding author upon request.
