# Supplementary material for: TREM2 deficiency in microglia accelerates photoreceptor cell death and immune cell infiltration following retinal detachment
Source: Cell Death Dis. 2023 Mar 28;14(3):219. doi: 10.1038/s41419-023-05735-x (PMC10050330; doi:10.1038/s41419-023-05735-x)
Supplement: Supplementary file 2 — Additional file 2 [file 41419_2023_5735_MOESM2_ESM.docx]

**Supplementary Information**

**Supplementary Figures**

**Figure S1.** **Acute RD leads to visual damage and impaired neuron communications at an early stage.**


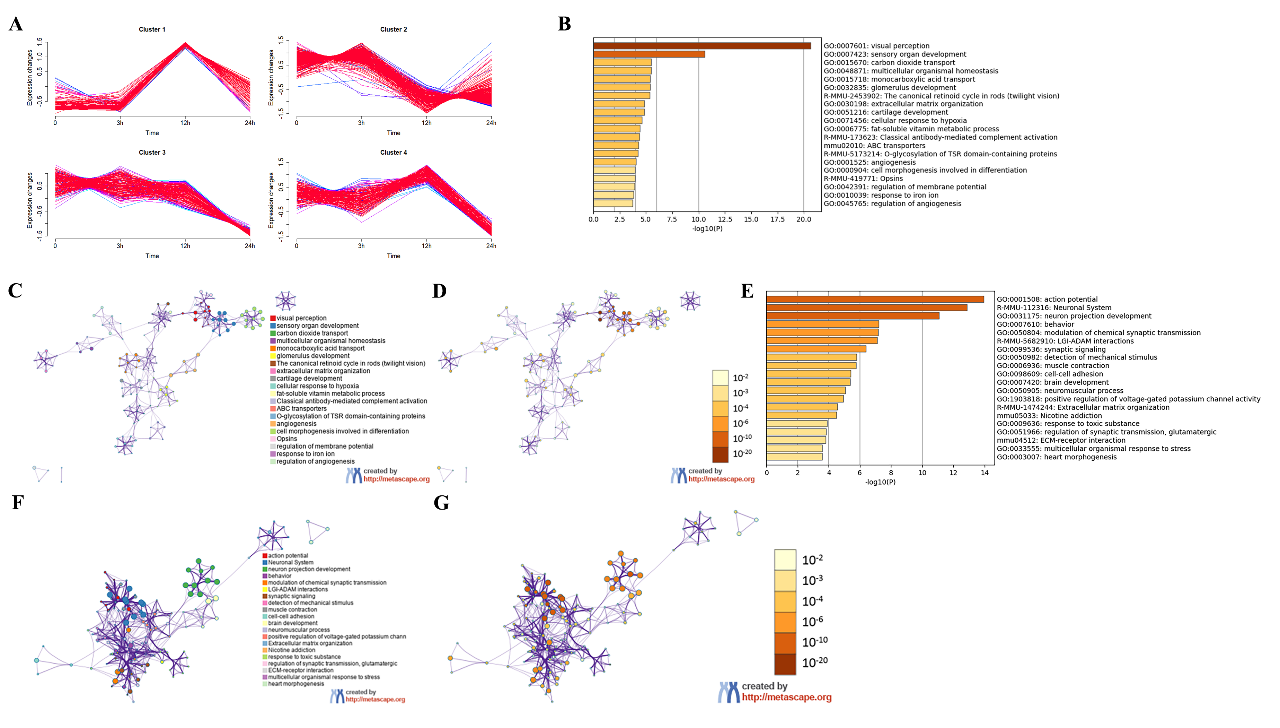


**Fig. S1** Acute RD leads to visual damage and impaired neuron communications at an early stage. **A** Time-course transcriptional profiles of down-regulated DEGs from the WT mouse RD model within 24 hrs. **B** The top 20 GO terms and pathways over-represented by the genes of Cluster 2. **C** Network of enriched terms colored by cluster ID in Cluster 2. **D** Network of enriched terms colored by *p*-value in Cluster 2. **E** The top 20 GO terms and pathways over-represented by the genes of Cluster 3. **F** Network of enriched terms colored by cluster ID in Cluster 3. **G** Network of enriched terms colored by *p*-value in Cluster 3.

**Figure S2. Representative immunofluorescent image of Ly6G positive cells in detached subretinal space of WT mouse 3d-post RD.**

**
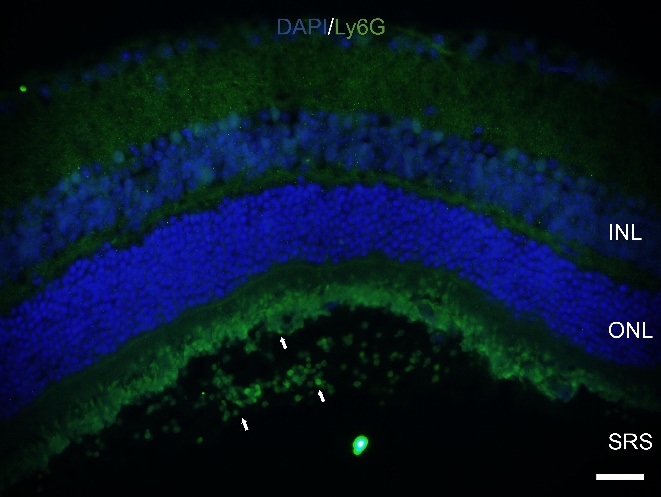
**

**Fig. S2** Representative immunofluorescent image of Ly6G staining showing the infiltrated neutrophils in the detached subretinal space. Scale bar, 50 μm.

**Figure S3.** **Representative immunofluorescent images of cleaved caspase 3 positive cells.**


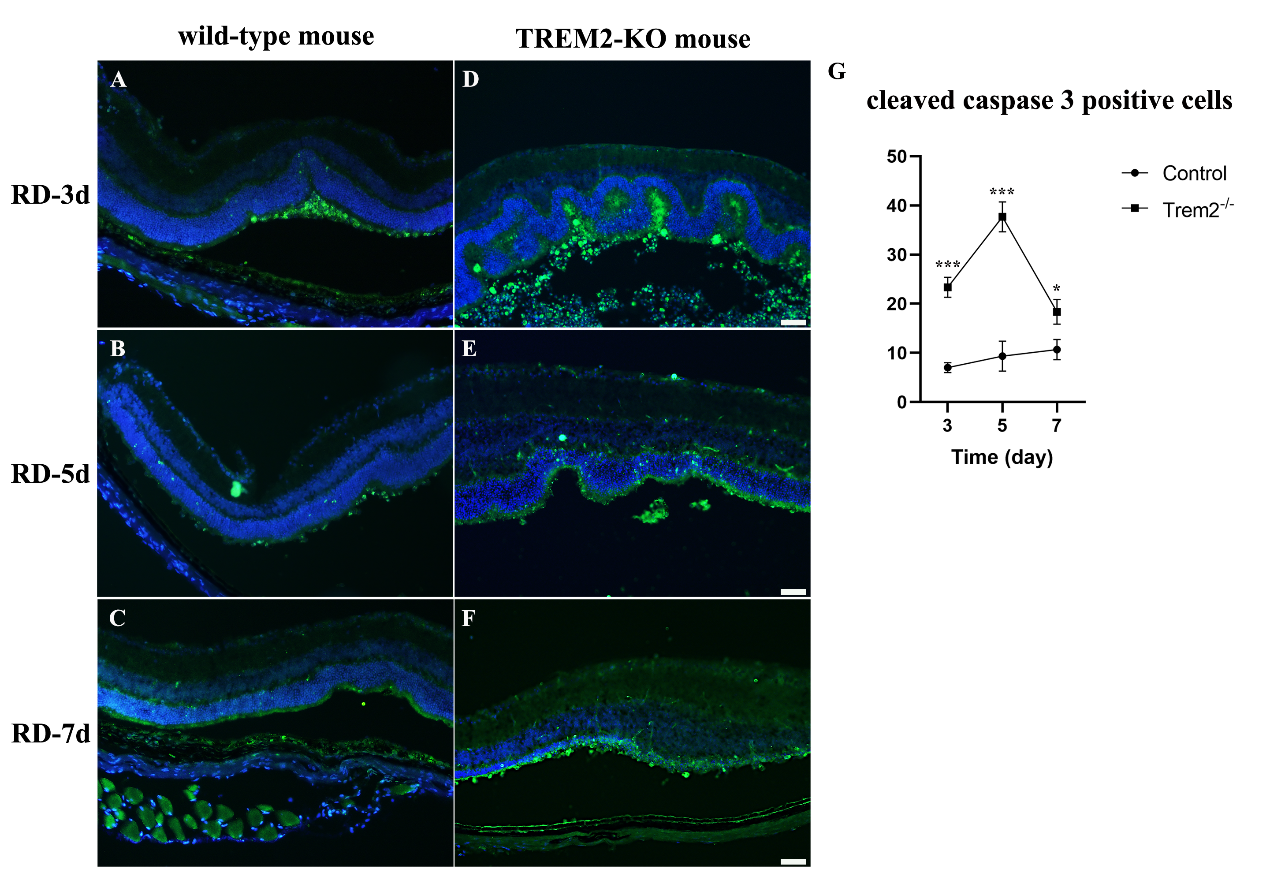


**Fig. S3** TREM2 deficiency accelerates photoreceptor apoptosis. **A-F** Representative immunofluorescent images of cleaved caspase 3 positive cells from 3 d to 7 d post-RD in wild-type (A-C) and *Trem2^-/-^* (D-F) mouse. Scale bar, 50 μm. **G** Quantitative analysis indicates that more photoreceptor cells were immunopositive for cleaved caspase-3 in Trem2^-/-^ mouse during RD. (n = 3 animals in each group). Column heights and error bars indicate mean ± SD, ns = not significant, **p* < 0.05, ***p* < 0.01, and ****p* < 0.001.

**Figure S4.** **The expression of homeostatic genes in retinal microglial cells sorted with CD11b MicroBeads.**


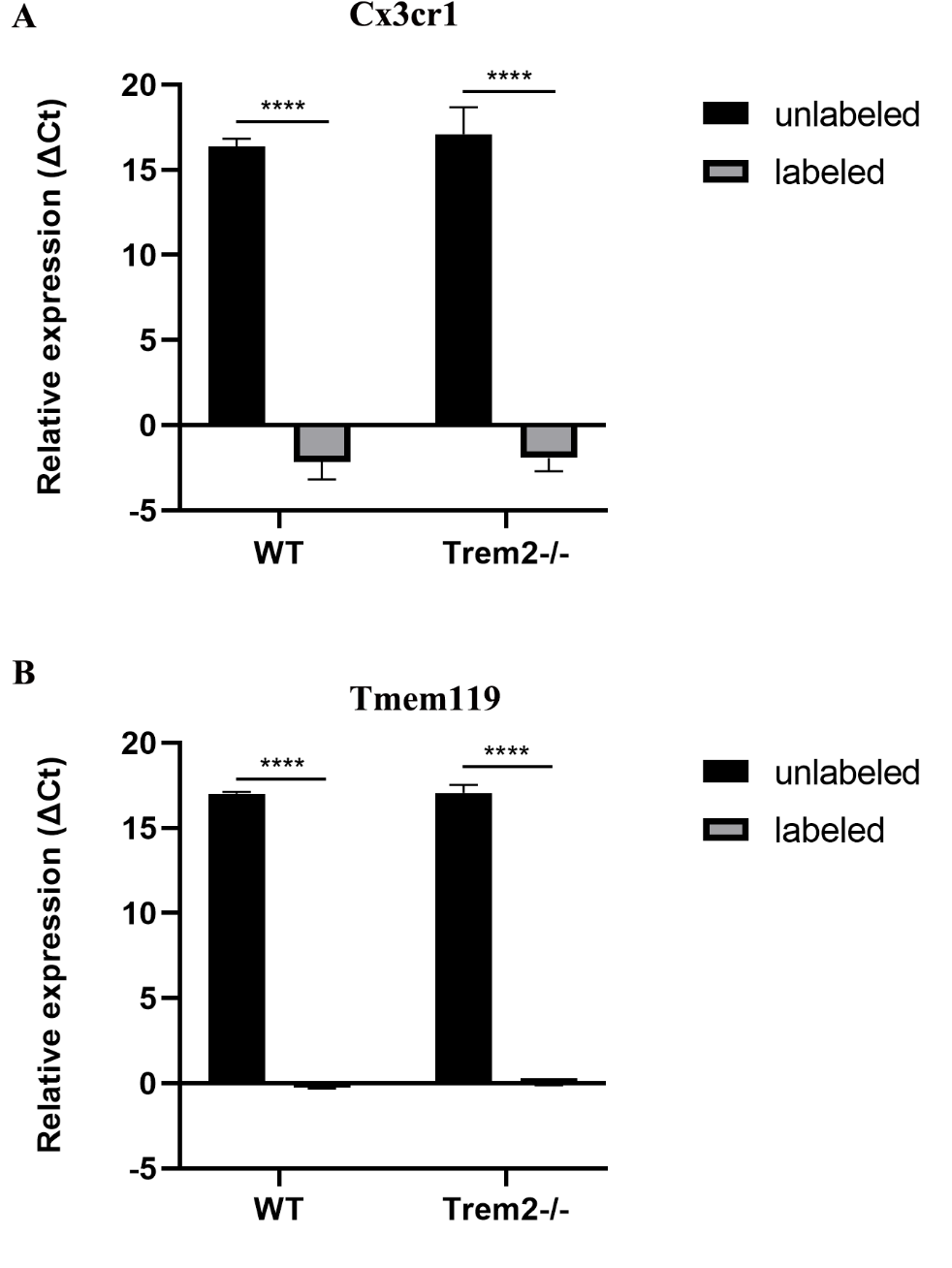


**Fig. S4** The expression of homeostatic genes in retinal microglial cells sorted with CD11b MicroBeads. **A** The expression of Cx3cr1 is increased in CD11b^+^ retinal cells sorted from WT and *Trem2^-/-^* mouse. **B** The expression of Tmem119 is increased in CD11b^+^ retinal cells sorted from WT and *Trem2^-/-^* mouse.

**Figure S5.** **The association between Ly6G^+^ and TUNEL^+^ cells on cross-sectioned 3 d post-RD retinal tissue.**


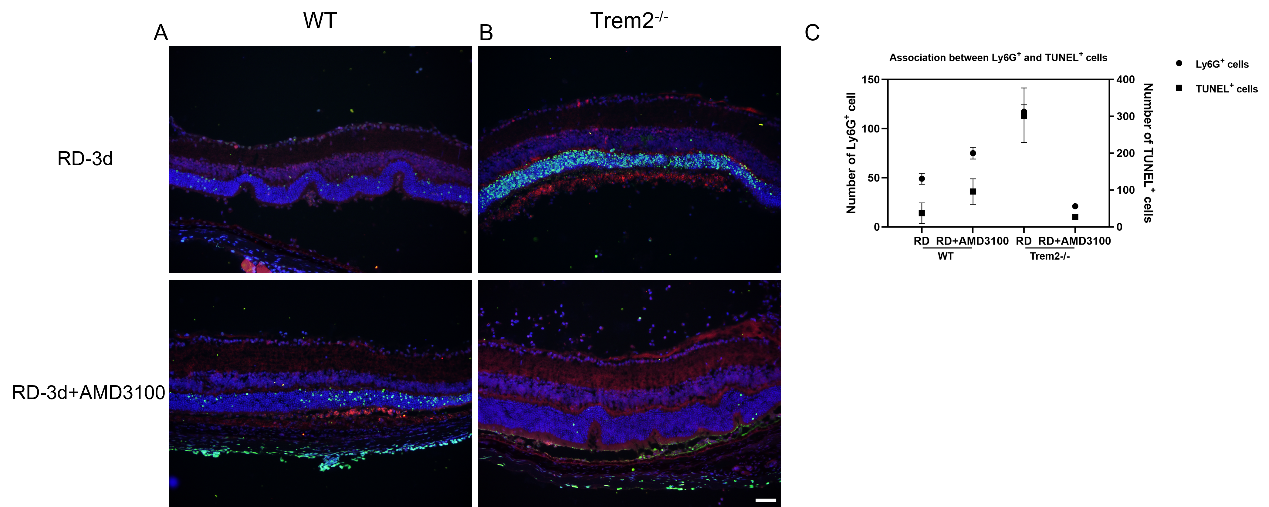


**Fig. S5** Representative immunofluorescent images of Ly6g^+^ and TUNEL^+^ cells on cross-sectioned 3 d post-RD retinal tissue in WT (**A**) and *Trem2^-/-^* (**B**) mouse. Scale bar, 50 μm. **C** Quantitative analysis indicates that as the number of TUNEL^+^ cells increased, more infiltrating neutrophils were recruited into the detached subretinal space. (n = 3 animals in each group).
